# Brain-only metastatic breast cancer as a distinct clinical entity: evidence from national and institutional cohorts with implications for radiation therapy

**DOI:** 10.1007/s10549-026-08092-3

**Published:** 2026-10-07

**Authors:** Marshall Harrell, Tugce Kutuk, Yevgeniya Gokun, Rituraj Upadhyay, Sachin R. Jhawar, Daniel Stover, Nicole Williams, Margaret E. Gatti-Mayes, Gina Sizemore, Heather LeFebvre, John C. Grecula, Raju R. Raval, Raj Singh, Simeng Zhu, Dukagjin M. Blakaj, Arnab Chakravarti, Joshua D. Palmer, Sasha J. Beyer

**Affiliations:** 1 Department of Radiation Oncology, The Ohio State University Wexner Medical Center, 460 W 10th Ave, Columbus, OH 43210 USA; 2 Center for Biostatistics, The Ohio State University Wexner Medical Center, Columbus, OH USA; 3 Department of Radiation Oncology, Moffitt Cancer Center, Tampa, FL USA; 4 Department of Medical Oncology, The Ohio State University Wexner Medical Center, Columbus, OH USA; 5 Department of Radiation Oncology, Lynn Cancer Institute, Baptist Health South Florida, Boca Raton, FL USA

**Keywords:** Breast cancer, Brain metastases, Stereotactic radiosurgery, Whole brain radiotherapy, National cancer database

## Abstract

**Purpose:**

Brain-only metastatic breast cancer (BO-MBC), defined as breast cancer with brain metastases (BrM) in the absence of extracranial metastatic disease (ECM), may represent a distinct metastatic phenotype. We characterized clinicopathologic features, treatment patterns, and outcomes of BO-MBC and evaluated associations between metastatic pattern, treatment, and survival.

**Methods:**

Patients with isolated BrM at our institution were evaluated for intracranial progression-free survival (iPFS), extracranial progression-free survival (ePFS), leptomeningeal disease-free survival (LMD-PFS), and overall survival (OS). Patients with MBC diagnosed between 2010 and 2020 were identified from the NCDB and classified as BO-MBC or BrM with concurrent ECM (BrM + ECM). Overlap propensity score-weighted Cox models evaluated associations between metastatic pattern, treatment, and OS.

**Results:**

In the institutional cohort (*n* = 30), intracranial progression was the predominant pattern of failure, with median iPFS of 14.6 months versus ePFS of 30.4 months. In the NCDB cohort (*n* = 8,909), 1,540 patients (17.3%) had BO-MBC. Compared with BrM + ECM, BO-MBC was associated with node-negative, triple-negative, and fewer HR+/HER2- disease (*p* < 0.001). BO-MBC patients were more likely to receive brain radiotherapy (54.8% vs. 41.0%, *p* < 0.001) but less likely to receive systemic therapy (67.2% vs. 74.5%, *p* < 0.001). In unadjusted Kaplan-Meier analyses, BO-MBC was associated with longer median OS than BrM + ECM (12.5 months [95% CI, 3.6–43.6] vs. 10.6 months [95% CI, 2.6–33.8]). Systemic therapy was associated with improved OS, with the greatest benefit in BO-MBC (adjusted HR 0.22, 95%CI 0.18–0.27). Brain radiotherapy was associated with improved OS in BO-MBC but not BrM + ECM.

**Conclusions:**

BO-MBC may represent a distinct clinical subgroup with unique clinicopathologic characteristics, treatment patterns, and improved survival. Intracranial progression predominated despite durable extracranial control, supporting optimized brain-directed management and prospective investigation of BO-MBC as a unique metastatic phenotype.

**Clinical trial number:**

Not applicable.

**Supplementary Information:**

The online version contains supplementary material available at https://doi.org/10.1007/s10549-026-08092-3.

## Introduction

Brain metastases (BrM) remain a major cause of morbidity and mortality in metastatic breast cancer (MBC), and their incidence appears to be increasing in the modern era [1]. The CNS is characterized as a sanctuary site that limits drug penetration, complicating both systemic therapy selection and intracranial disease control [2]. Despite advances in multidisciplinary management, including neurosurgery, radiotherapy, and CNS-penetrant systemic therapies, outcomes for many patients with BrM remain poor, particularly for those with diffuse intracranial disease or leptomeningeal disease (LMD).

Within this broader population, a clinically important subset of patients presents with brain-only MBC (BO-MBC), defined by BrM in the absence of extracranial metastases (ECM). BO-MBC is increasingly recognized as a potential distinct clinical phenotype rather than simply a transitional stage of disseminated metastatic disease. Prior institutional and multicenter retrospective series suggest BO-MBC may be enriched for HER2 + and triple-negative subtypes, younger age, and more limited intracranial burden (often solitary or oligometastatic disease) [3, 4]. Notably, early reports have suggested longer survival for BO-MBC compared with patients presenting with concurrent ECM, raising the possibility that the metastatic pattern itself conveys prognostic information and may inform management strategy [3, 5, 6]. However, BO-MBC remains understudied and direct comparisons between BO-MBC and BrM + ECM cohorts in large contemporary datasets remain limited.

Current management principles for breast cancer BrM reflect a shift toward individualized intracranial treatment strategies. WBRT, historically used for patients with multiple or diffuse metastases, is increasingly reserved for select scenarios, as growing evidence supports the use of stereotactic radiosurgery/stereotactic radiotherapy (SRS/SRT) to preserve neurocognitive function while maintaining intracranial control [7]. The ability to perform repeat SRS/SRT for salvage further strengthens its role in patients with CNS-predominant disease. Concurrently, advancements in systemic therapy including CNS-penetrant HER2-targeted agents, antibody-drug conjugates, CDK4/6 inhibitors for HR-positive disease, and novel therapies for triple-negative breast cancer have expanded the therapeutic toolbox [2]. These developments reinforce the importance of optimizing local therapy while ensuring effective systemic disease management, a balance that may be particularly relevant for BO-MBC, where the trajectory of disease may be driven predominantly by intracranial rather than extracranial progression.

To address persistent gaps in evidence, we performed an institutional and national cancer database (NCDB) analysis of patients with MBC. First, we evaluated an institutional cohort of patients with BO-MBC to characterize clinicopathologic features, treatment patterns, and CNS-specific outcomes, including intracranial progression-free survival (iPFS), extracranial progression-free survival (ePFS), leptomeningeal disease-free survival (LMD-PFS), and overall survival (OS). We then analyzed patients with MBC in the NCDB diagnosed between 2010 and 2020 to evaluate clinicopathologic characteristics, treatment patterns, and OS in patients with BO-MBC compared with those with BrM + ECM. Finally, among patients receiving brain radiotherapy, we evaluated the association between radiotherapy modality (WBRT versus SRS/SRT) and survival outcomes.

## Methods

### Institutional cohort

Breast cancer patients with BO-MBC, defined as synchronous or metachronous BrM, in the absence of ECM, were identified from our institutional database between 1/1/2017- 7/31/2024. Patients with leptomeningeal disease at the time of BrM diagnosis were excluded. Demographics, primary tumor receptor profile, receptor profile of BrM when available, and treatment details, including radiotherapy approach, were abstracted from the medical record. Time-to-event outcomes were measured from the date of BrM diagnosis and included iPFS, ePFS, LMD-PFS, and OS. LMD-related outcomes were evaluated exclusively in the institutional cohort because the NCDB does not capture LMD or progression-related outcomes. Intracranial progression was defined as parenchymal progression and/or the subsequent development of LMD. Progression events were determined from imaging and clinical documentation. Kaplan-Meier methods were used to estimate survival distributions, with censoring at last follow-up. The institutional study was approved by the local Institutional Review Board with waiver of informed consent.

### NCDB cohort

**Fig. 1:**
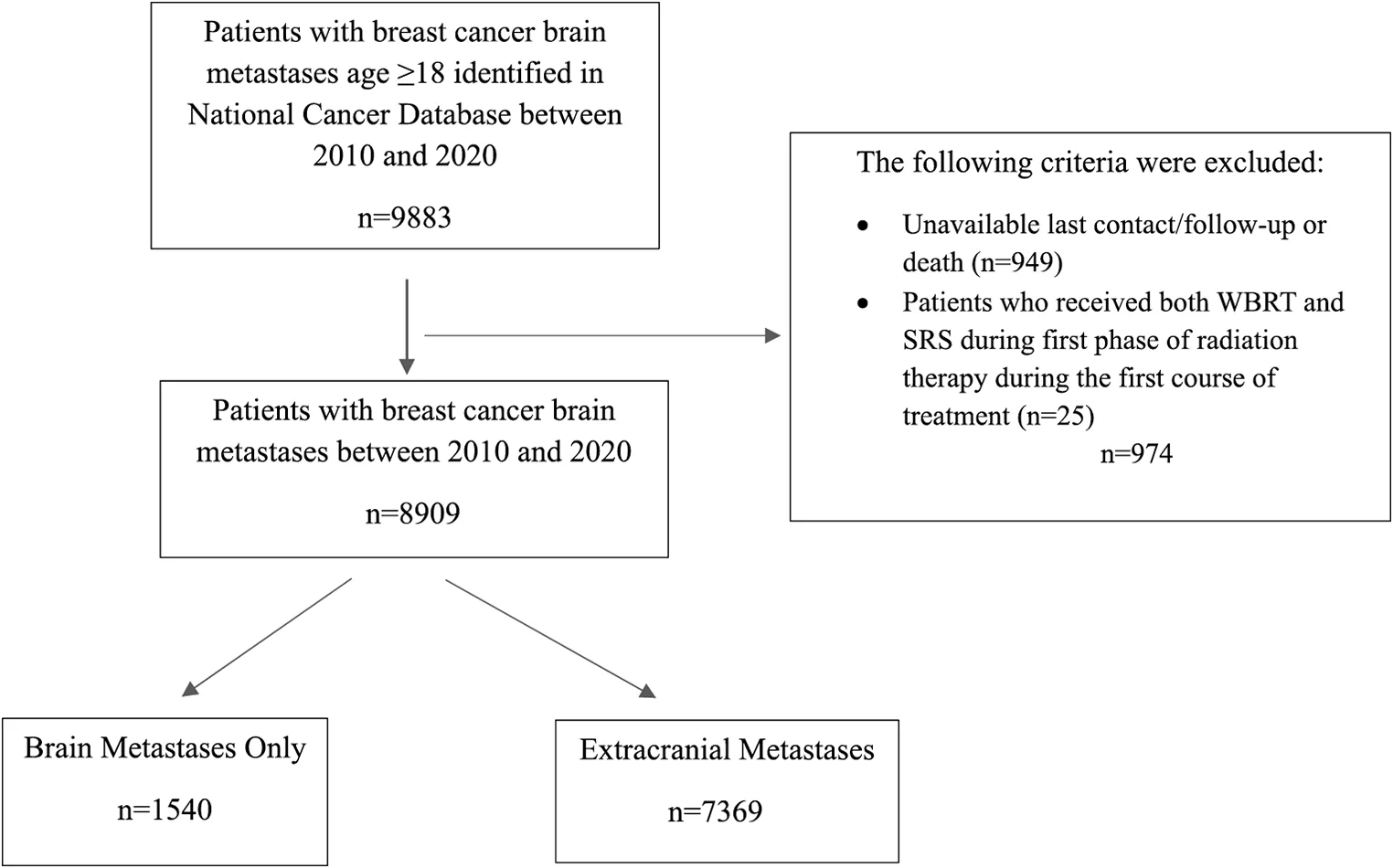
Study schema

Data for this study were obtained from the NCDB, a nationwide, hospital-based cancer registry jointly sponsored by the American College of Surgeons and the American Cancer Society, capturing approximately 70% of all newly diagnosed malignancies across more than 1,500 Commission on Cancer (CoC)-accredited facilities in the United States [8]. Patients aged ≥ 18 years with breast cancer and BrM at diagnosis, diagnosed between 2010 and 2020, were identified in the NCDB (Fig. 1). Metastatic-site variables in the NCDB capture metastases present at the time of initial breast cancer diagnosis. Accordingly, the NCDB cohort was restricted to patients with de novo MBC and synchronous BrM and did not include patients who subsequently developed metachronous BrM after an earlier-stage diagnosis. To complement the institutional cohort and evaluate the generalizability of our findings, we performed a parallel analysis comparing clinicopathologic characteristics, treatment patterns, and overall survival between patients with BO-MBC and those with brain metastases and concurrent extracranial metastatic disease (BrM + ECM). Sociodemographic variables included age at diagnosis, race, and ethnicity. Clinical variables included the Charlson-Deyo score (CDS), molecular subtype, receipt of systemic therapy, and receipt of brain radiotherapy. The CDS, as defined in the NCDB codebook, is a summary index captured at diagnosis and categorized as 0, 1, 2, or ≥ 3 comorbid conditions. Molecular subtype was defined based on hormone receptor (estrogen and/or progesterone receptor) and HER2 status as follows: HR+/HER2−, HR+/HER2+, HR−/HER2+, and HR−/HER2−. Systemic therapy was defined as receipt of one or more modalities, including chemotherapy, endocrine therapy, and/or immunotherapy. The primary exposure was metastatic group type, dichotomized based on metastatic pattern at diagnosis: BO-MBC versus BrM + ECM. The secondary exposure was type of brain radiotherapy received: WBRT versus SRS/SRT. For NCDB analyses, the primary endpoint was OS, defined as the time from diagnosis to death from any cause or last known follow-up, whichever occurred first. iPFS, ePFS, LMD-PFS were not available in the NCDB and therefore evaluated only in the institutional cohort.

### Statistical analysis

Descriptive statistics were used to summarize baseline characteristics. Continuous variables were reported as medians with interquartile ranges (IQRs), and categorical variables were summarized using frequencies and proportions. Differences between BO-MBC and BrM + ECM groups were assessed using the Wilcoxon rank-sum test for continuous variables and the chi-square test for categorical variables. Median OS and 95% confidence intervals were estimated using unadjusted Kaplan-Meier analyses.

Overlap propensity score weighting (OPSW) was used to adjust for confounding due to baseline differences between groups [9, 10]. Propensity scores were estimated using multivariable logistic regression, with group type (BO-MBC vs. BrM + ECM) as the exposure and age, race, ethnicity, CDS, molecular subtype, systemic therapy, and brain radiotherapy as covariates. These variables were selected a priori based on clinical relevance and potential associations with group type and OS. Covariate balance after weighting was assessed using standardized differences (Supplementary Table 1), defined as the absolute difference in means or proportions divided by the pooled standard deviation. A standardized difference < 0.1 was considered indicative of adequate balance [11]. Separate OPSW Cox proportional hazards models with robust variance were used to evaluate associations between group type and OS, including interaction terms for group type × brain radiotherapy and group type × systemic therapy.

As an exploratory analysis, patients receiving brain radiotherapy were stratified by radiotherapy modality (SRS/SRT vs. WBRT) within the BO-MBC and BrM + ECM cohorts. Propensity scores for radiotherapy modality were estimated using age, race, ethnicity, CDS, insurance status, community type, molecular subtype, treatment facility type, chemotherapy, immunotherapy, and hormone therapy. Balance between groups was assessed using standardized differences (Supplementary Table 2). All analyses were performed using SAS version 9.4 (SAS Institute Inc.). Two-sided tests were considered statistically significant at a significance level of 0.05.

## Results

### Institutional cohort

The institutional cohort comprised 30 patients with BO-MBC, with a median follow-up of 26.5 months after BrM diagnosis. Primary tumor subtypes were HR+/HER2- (*n* = 5, 16.7%), HR+/HER2+ (*n* = 6, 20.0%), HR-/HER2+ (*n* = 9, 30.0%), and triple-negative (*n* = 10, 33.3%), with HER2-positive tumors accounting for 50.0% of the cohort. Most patients were symptomatic at presentation (93.3%), most commonly with headaches. The median number of BrM at diagnosis was 2 (range, 1–41), with a median total intracranial tumor volume of 15.1 cc (Table 1). Of the 25 (83.3%) patients who underwent neurosurgical resection, pathology of the BrM was HER2 + in 12 (48%), triple-negative in 9 (36%), and HR + in 8 (32%). Receptor discordance between the primary breast tumor and BrM was observed in 7 patients (28%). All discordant events involved HR status, while no HER2 discordance was observed. Overall HR subtype conversion occurred in 5 patients, including 3 patients who converted from HR-positive to HR-negative disease and 2 who converted from HR-negative to HR-positive disease. The remaining 2 discordant cases reflected isolated loss of PR expression without alteration in overall HR status. All patients received brain radiotherapy, including SRS/SRT in 86.7% (median dose 24 Gy in 3 fractions) and WBRT in 13.3% (median dose 30 Gy in 10 fractions). New systemic therapy was initiated at the time of BrM diagnosis in 66.7% of patients (*n* = 20), while 5 patients (16.7%) continued their existing systemic regimen. Thus, 25 patients (83.3%) received systemic therapy at the time of BrM diagnosis, whereas 5 patients (16.7%) did not receive systemic therapy within 3 months after BrM diagnosis. In 3 patients, this reflected patient preference; in 1 patient with brain-only metastatic TNBC, the multidisciplinary tumor board recommended surveillance without systemic chemotherapy. The remaining patient subsequently developed sacral metastasis followed by LMD and initiated intrathecal chemotherapy more than 3 months after BrM diagnosis.

**Table 1:** Baseline clinicopathologic characteristics and treatment patterns of patients with brain-only metastatic breast cancer in the institutional and NCDB cohorts

Variable	Institutional BO-MBC (*n* = 30)	NCDB BO-MBC (*n* = 1,540)
**Demographics**		
**Age at MBC diagnosis**, median (IQR)	51 (39–59)	62 (52–71)
**Female sex**, n (%)	30 (100%)	N/A
**Race**, n (%)		
White	24 (80.0%)	1,142 (75.3%)
Black	6 (20.0%)	298 (19.6%)
Asian	0 (0%)	50 (3.3%)
Other	0 (0%)	27 (1.8%)
**Ethnicity**, n (%)		
Non-Hispanic	30 (100%)	1361 (88.4%)
Hispanic	0 (0%)	129 (8.4%)
Unknown	N/A	50 (3.2%)
**Tumor Biology**		
**Histology**, n (%)		
IDC	27 (90.0%)	938 (60.9%)
ILC	1 (3.3%)	121 (7.9%)
Other/mixed/unknown	2 (6.7%)	481 (31.2%)
**Molecular subtype**, n (%)		
HR+/HER2-	5 (16.7%)	342 (22.2%)
HR+/HER2+	6 (20.0%)	176 (11.4%)
HR-/HER2+	9 (30.0%)	173 (11.2%)
Triple Negative	10 (33.3%)	400 (26.0%)
Unknown	0 (0%)	449 (29.2%)
**Clinical Stage at Initial Diagnosis**		
**Clinical T stage**, n (%)		
T0	0 (0%)	57 (3.7%)
T1	11 (36.7%)	246 (16.0%)
T2	9 (30.0%)	390 (25.3%)
T3	7 (23.3%)	161 (10.5%)
T4	2 (6.7%)	315 (20.5%)
Unknown	1 (3.3%)	371 (24.1%)
**Clinical N stage**, n (%)		
N0	16 (53.3%)	439 (28.5%)
N1	11 (36.7%)	495 (32.1%)
N2	0 (0%)	123 (8.0%)
N3	2 (6.7%)	208 (13.5%)
Unknown	1 (3.3%)	275 (17.9%)
**Treatment Patterns**		
**Systemic therapy**, n (%)		
Yes	25 (83.3%)	1033 (67.2%)
No	5 (16.7%)	504 (32.8%)
**Neoadjuvant chemotherapy**, n (%)	19 (63.3%)	118 (7.7%)
**Brain radiotherapy**, n (%)		
Yes	30 (100.0%)	844 (54.8%)
No	0 (0.0%)	696 (45.2%)
**Neurosurgical resection**, n (%)	25 (83.3%)	N/A
**Brain Metastasis Characteristics**		
**Number of brain metastases**, median (range)	2 (1–41)	N/A
**Total intracranial tumor volume**, median cc (range)	15.1 (0.4–99.4)	N/A
**Symptomatic at presentation**, n (%)	28 (93.3%)	N/A

Abbreviations: BO-MBC, brain-only metastatic breast cancer; HR, hormone receptor; HER2, human epidermal growth factor receptor 2.Unknown categories reflect missing registry data in the NCDB

At a median follow-up of 26.5 months following BrM diagnosis, median OS was not reached. One- and 2-year OS rates were 97% and 90%, respectively, while OS at 30.6 months was 65% (95% CI, 39–82%). Median iPFS was 14.6 months, and median ePFS was 30.4 months. LMD-PFS was not reached, with a 28.3-month LMD-PFS rate of 75% (95% CI, 52–89%). Intracranial progression, defined as parenchymal progression and/or development of LMD, occurred in 20 patients (66.7%). Six of these patients (20.0% of the entire cohort) developed LMD. Extracranial progression occurred in 14 patients (46.7%). Outcomes of the institutional cohort are further summarized in Table 2.

**Table 2:** Institutional cohort: progression patterns, survival outcomes, and cns-related toxicity (n = 30)

	Total patients (n = 30)
**Follow-up**	
Median follow-up after BrM diagnosis, months (IQR; range)	26.5 (15.8-34.1; 6.2-144.5)
**Overall Survival**	
Median overall survival, months	Not reached
Overall survival at 1 year, %	97%
Overall survival at 2 years, %	90%
Overall survival at 30.6 months, % (95% CI)	65% (95% CI 39-82%)
**Patterns of Progression**	
Median intracranial progression-free survival (iPFS), months	14.6
Median extracranial progression-free survival (ePFS), months	30.4
Median leptomeningeal disease-free survival (LMD-PFS), months	Not reached
LMD-PFS at 28.3 months, % (95% CI)	75% (95% CI 52-89%)
Intracranial progression, n (%)	20 (66.7%)
Extracranial progression, n (%)	14 (46.7%)
Leptomeningeal disease (LMD), n (%)	6 (20.0%)
**CNS-Directed Therapy**	
SRS/FSRT, n (%)	26 (86.7%)
WBRT, n (%)	4 (13.3%)
Median dose, Gy (IQR; range)	24 (24-25; 18-30)
Median number of fractions	3
**Systemic Therapy**	
Initiation of new systemic therapy at brain metastasis diagnosis, n (%)	20 (66.7%)
Continuation of existing systemic therapy at brain metastasis diagnosis, n (%)	5 (16.7%)
Neoadjuvant chemotherapy prior to breast surgery, n (%)	19 (63.3%)
**Treatment-Related Toxicity**	
Radiation necrosis, n (%)	13 (43.3%)
**Vital Status**	
Alive at last follow-up	23 (76.7%)
Deaths, n (%)	7 (23.3%)
Neurologic cause of death among deaths, n (%)	7 (100%)

Abbreviations: BrM, brain metastases; iPFS, intracranial progression-free survival; ePFS, extracranial progression-free survival; LMD, leptomeningeal disease; SRS, stereotactic radiosurgery; FSRT, fractionated stereotactic radiotherapy; WBRT, whole-brain radiotherapy

Median overall survival and median leptomeningeal disease-free survival were not reached due to limited events

### NCDB cohort

Baseline clinicopathologic characteristics of the NCDB cohort are summarized in Table 3. Among 8,909 patients with MBC, 1,540 (17.3%) had BO-MBC and 7,369 (82.7%) had BrM + ECM. BO-MBC demonstrated distinct clinicopathologic characteristics compared with BrM + ECM. Patients with BO-MBC were slightly older at diagnosis (median age 62 vs. 61 years, *p* = 0.011) and demonstrated a higher proportion of clinically node-negative disease (N0: 28.5% vs. 20.1%, *p* < 0.001) and grade 3 tumors (33.0% vs. 30.5%, *p* < 0.001). Additionally, patients with BO-MBC were less likely to have HR+/HER2 − disease (22.2% vs. 31.3%) and more likely to have triple-negative tumors (26.0% vs. 17.7%) (*p* < 0.001). The distribution of HER2-positive subtypes was similar between groups, with only modest differences in HR+/HER2+ (11.4% vs. 13.8%) and HR−/HER2 + disease (11.2% vs. 10.4%).

**Table 3:** Sociodemographic, clinical, and treatment characteristics comparing brain-only and extracranial metastatic breast cancer in the NCDB cohort (*n* = 8,909)

Variable	BO-MBC (*n* = 1,540)	BrM + ECM (*n* = 7,369)	*P*-value
**Age**, median (IQR)	62 (52–71)	61 (53–69)	0.011
**Molecular Subtype**			< 0.001
HR+/HER2-	342 (22.2%)	2305 (31.3%)	
HR+/HER2+	176 (11.4%)	1014 (13.8%)	
HR-/HER2+	173 (11.2%)	765 (10.4%)	
Triple negative	400 (26.0%)	1305 (17.7%)	
Unknown	449 (29.2%)	1980 (26.9%)	
**Clinical N stage**,** n (%)**			< 0.001
N0	439 (28.5%)	1480 (20.1%)	
N1	495 (32.1%)	2803 (38.0%)	
N2	123 (8.0%)	741 (10.1%)	
N3	208 (13.5%)	952 (12.9%)	
Unknown	275 (17.9%)	1393 (18.9%)	
**Tumor grade**, n (%)			< 0.001
Grade 1	73 (4.7%)	301 (4.1%)	
Grade 2	274 (17.8%)	1652 (22.4%)	
Grade 3	508 (33.0%)	2259 (30.5%)	
Unknown	685 (44.5%)	3166 (43.0%)	
**Systemic therapy**,** n (%)**			< 0.001
Yes	1033 (67.2%)	5483 (74.5%)	
No	504 (32.8%)	1875 (25.5%)	
**Brain radiotherapy**, n (%)			< 0.001
Yes	844 (54.8%)	3020 (41.0%)	
No	696 (45.2%)	4349 (59.0%)	

Abbreviations: HR, hormone receptor; HER2, human epidermal growth factor receptor 2.Models were adjusted for age, race, ethnicity, Charlson-Deyo comorbidity score, insurance status, community type, treatment facility type, molecular subtype, and receipt of chemotherapy, hormone therapy, and immunotherapy. These variables are presented in Supplemental Table 1

Treatment patterns also varied substantially between cohorts. Patients with BO-MBC were more likely to receive brain radiotherapy (54.8% vs. 41.0%, *p* < 0.001), while systemic therapy utilization was lower compared with BrM + ECM patients (67.2% vs. 74.5%, *p* < 0.001). Among patients receiving brain radiotherapy, patients with BO-MBC were more likely to receive SRS/SRT than patients with BrM + ECM (25.3% vs. 20.6%, *p* = 0.0023). OPSW demonstrated adequate covariate balance between metastatic groups, with standardized differences approaching zero after weighting (Supplemental Table 1). Among patients receiving brain radiotherapy, those treated with SRS/SRT were more frequently managed at academic centers (40.4% vs. 32.9%, *p* < 0.001) and more likely to receive systemic therapy (86.8% vs. 81.1%, *p* < 0.001) compared with patients receiving WBRT (Supplemental Table 3).

Increasing age and higher CDS were independently associated with worse OS (Table 4). Molecular subtype was also prognostic, with triple-negative subtype associated with significantly higher mortality relative to HR+/HER2 − disease (adjusted HR 1.76, 95% CI 1.60–1.95), whereas HR+/HER2 + disease demonstrated improved OS (adjusted HR 0.86, 95% CI 0.76–0.98). Receipt of systemic therapy was also strongly associated with improved OS (adjusted HR 0.42, 95% CI 0.38–0.46). In unadjusted Kaplan-Meier analyses, median OS was 12.5 months (95% CI, 3.6–43.6) in the BO-MBC group and 10.6 months (95% CI, 2.6–33.8) in the BrM + ECM group.

**Table 4:** Multivariable cox regression analysis of overall survival in the NCDB cohort (*n* = 8,909)

Variable	Adjusted Hazard Ratio (95% CI)
**Age**	
Per 1-year increase	1.014 (1.011–1.017)
**Race**	
White	Ref
African American/Black	1.05 (0.96–1.15)
Asian	0.88 (0.71–1.08)
Other	0.69 (0.50–0.96)
**Ethnicity**	
Non-Hispanic	Ref
Hispanic	0.72 (0.62–0.83)
**Charlson-Deyo Score**	
0	Ref
1	1.16 (1.04–1.30)
2	1.29 (1.10–1.52)
3+	1.39 (1.06–1.84)
**Molecular subtype**	
HR+/HER2-	Ref
HR+/HER2+	0.86 (0.76–0.98)
HR-/HER2+	1.08 (0.96–1.22)
Triple-negative	1.76 (1.60–1.95)
Unknown	1.29 (1.17–1.42)
**Systemic therapy**	
Yes	0.42 (0.38–0.46)
No	Ref
**Interaction: Metastatic pattern x Brain RT**	
BO-MBC with Brain RT	0.67 (0.58–0.77)
BO-MBC with No Brain RT	0.69 (0.61–0.77)
BrM + ECM with Brain RT	0.97 (0.91–1.03)
BrM + ECM with No Brain RT	Ref

HR=hazard ratio, CI=confidence interval

**Fig. 2:**
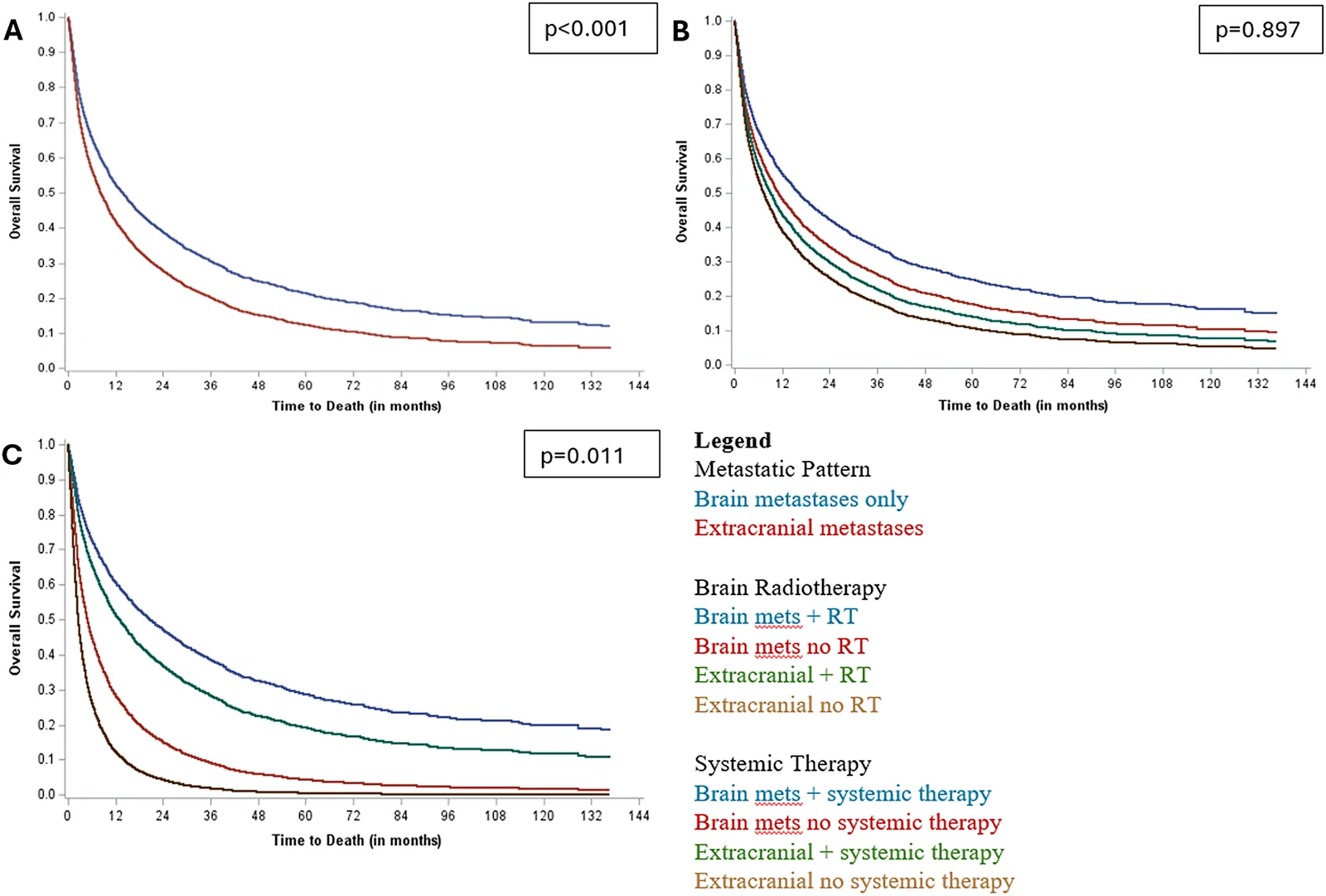
Overall survival comparing metastatic pattern and treatment utilization in the NCDB cohort (n=8,909) *. Kaplan-Meier curves demonstrate OS according to (**A**) metastatic pattern, comparing BO-MBC with BrM+ECM; (**B**) metastatic pattern and receipt of brain radiotherapy, comparing BO-MBC with radiotherapy, BO-MBC without radiotherapy, BrM+ECM with radiotherapy, and BrM+ECM without radiotherapy; and (**C**) metastatic pattern and receipt of systemic therapy, comparing the corresponding four groups. The P value in panel A represents the log-rank comparison between metastatic groups, whereas the P values in panels B and C represent interaction tests between metastatic pattern and receipt of brain radiotherapy and systemic therapy, respectively. Abbreviations: BO-MBC, brain-only metastatic breast cancer; BrM+ECM, brain metastases with concurrent extracranial metastases; NCDB, National Cancer Database; OS, overall survival; RT, radiotherapy

Overall, patients with BO-MBC demonstrated improved OS compared with patients with BrM + ECM (Fig. 2A). This OS advantage remained evident regardless of receipt of brain radiotherapy (Fig. 2B). Relative to the BrM + ECM with no brain radiotherapy reference group, BO-MBC exhibited lower hazards of death both among patients who received brain radiotherapy (adjusted HR 0.67, 95% CI 0.58–0.77) and among those who did not (adjusted HR 0.69, 95% CI 0.61–0.77). In contrast, among BrM + ECM patients, receipt of brain radiotherapy was not associated with a statistically significant OS difference relative to BrM + ECM with no brain radiotherapy (adjusted HR 0.97, 95% CI 0.91–1.03). Receipt of systemic therapy was associated with improved OS across metastatic patterns (Fig. 2C). Compared with BrM + ECM with no systemic therapy, BO-MBC patients receiving systemic therapy had the most favorable outcomes (adjusted HR 0.22, 95% CI 0.18–0.27), while BO-MBC patients who did not receive systemic therapy also demonstrated improved OS (adjusted HR 0.60, 95% CI 0.51–0.70). Among BrM + ECM patients, systemic therapy remained strongly associated with improved OS (adjusted HR 0.38, 95% CI 0.35–0.41).

Within BO-MBC, patients receiving brain radiotherapy were younger (median 60 vs. 63 years, *p* = 0.0001) and more frequently received systemic therapy (79.0% vs. 52.9%, *p* < 0.0001). After adjustment for age, race, ethnicity, Charlson-Deyo score, insurance status, community type, molecular subtype, treatment facility type, chemotherapy, immunotherapy, and hormone therapy, SRS/SRT was associated with improved OS compared with WBRT in both groups (BO-MBC: aHR, 0.76; 95% CI, 0.62–0.93; BrM + ECM: aHR, 0.77; 95% CI, 0.70–0.86).

## Discussion

Our institutional cohort findings were consistent with prior series, in which intracranial progression remained common in patients with BO-MBC despite aggressive local therapy, with published brain-specific PFS typically ranging from 4 to 9 months and LMD representing a key failure mode [4, 12–14]. In contrast, durable extracranial disease control appears to be a defining feature of this population, particularly in HER2 + disease, and is mirrored by the prolonged ePFS observed in this cohort [4]. Reported OS for BO-MBC (approximately 11–21 months, with a meaningful subset of long-term survivors) aligns closely with the outcomes observed here [4, 6, 13]. Notably, the incidence of LMD progression in this cohort parallels prior published experience, underscoring the ongoing challenge of intracranial disease control in patients who may live long enough for neurologic progression to substantially impact quality of life and functional outcomes [13, 14].

In the national cohort of MBC patients, BO-MBC was associated with differences in recorded clinicopathologic characteristics and improved OS compared with patients presenting with BrM + ECM. These observations raise, but do not establish, the possibility that BO-MBC represents a distinct metastatic phenotype and should be interpreted in the context of missing registry data. In the NCDB cohort, BO-MBC was more frequently associated with clinically node-negative disease at diagnosis (28.5% vs. 20.1%) and had a higher proportion of triple-negative tumors (26.0% vs. 17.7%), with a correspondingly lower proportion of HR+/HER2 − disease (22.2% vs. 31.3%). Although the NCDB does not capture intracranial tumor burden, CNS-specific genomics, or receptor discordance, these patterns are directionally consistent with prior series suggesting that baseline disease extent and tumor biology may influence metastatic trajectories and outcomes [15–17]. The subtype distribution in the NCDB cohort demonstrated enrichment of triple-negative disease in BO-MBC, whereas differences in HER2 + tumors appear comparatively modest. In contrast, our institutional cohort demonstrated a higher proportion of HER2 + disease, consistent with prior reports suggesting enrichment of HER2 + and triple-negative subtypes among patients with BO-MBC, including data from Id Said et al., which demonstrated higher odds of these subtypes in BO-MBC compared with HR+/HER2- disease [4]. More broadly, studies of breast cancer BrM, though not restricted to BO-MBC, have shown overrepresentation of triple-negative, and HER2 + tumors relative to unselected breast cancer populations, further supporting a CNS-tropic phenotype [18]. From a radiation oncology standpoint, these findings are clinically important because patients with BO-MBC may have fewer competing extracranial risks and may live long enough for intracranial control, salvage options, and late effects of CNS-directed therapy to become dominant determinants of quality of life and function. These clinicopathologic differences should, however, be interpreted cautiously. Molecular subtype was unknown in 29.2% of patients with BO-MBC and 26.9% of those with BrM + ECM; tumor grade was unknown in 44.5% and 43.0%, and clinical nodal status in 17.9% and 18.9%, respectively. This degree of missingness may have influenced the observed distributions and limits conclusions regarding underlying biologic differences. Furthermore, because the NCDB does not capture recurrence, this analysis was restricted to patients with de novo MBC and may not be generalizable to those who develop metachronous brain metastases. Although BO-MBC was associated with higher recorded proportions of triple-negative and grade 3 disease, triple-negative disease remained independently associated with worse OS. Thus, the improved OS observed with BO-MBC may reflect the absence of concurrent extracranial disease rather than more favorable tumor biology. Residual confounding from missing or unmeasured prognostic factors cannot be excluded.

Our findings highlight that treatment associations in MBC are strongly conditioned by metastatic pattern. Patients with BO-MBC were more likely to receive brain radiotherapy yet less likely to receive systemic therapy than those with extracranial disease, a pattern consistent with prior reports and suggestive of continued reliance on locoregional CNS-directed therapy in the absence of overt systemic spread. However, in adjusted analyses, BO-MBC remained associated with improved overall survival compared with ECM, and receipt of systemic therapy was strongly associated with improved survival, with the most favorable outcomes observed among BO-MBC patients receiving systemic therapy. Interaction analyses further demonstrated that the associations between treatment exposures and survival differed by metastatic pattern, underscoring that treatment effects cannot be interpreted independently of disease distribution.

Importantly, the majority of patients included in the present NCDB cohort were diagnosed prior to the widespread integration of contemporary CNS-active systemic therapies and the modern multidisciplinary treatment paradigm for breast cancer with BrM. Consequently, the observed association between systemic therapy utilization and improved OS in this analysis likely underestimates the prognostic impact achievable in the current therapeutic era, particularly among patients with BO-MBC who may experience prolonged OS due to limited ECM burden. Over the past several years, multiple agents with clinically meaningful intracranial activity have significantly altered the treatment landscape of MBC. In HER2 + disease, the HER2CLIMB trial demonstrated that the addition of tucatinib to trastuzumab and capecitabine significantly improved OS and iPFS in patients with active BrM, with an intracranial objective response rate of 47.3% [19]. Similarly, trastuzumab deruxtecan (T-DXd) has demonstrated substantial intracranial efficacy across multiple studies in HER2 + MBC with BrM. In the phase II TUXEDO-1 trial, T-DXd achieved an intracranial objective response rate of 73.3% in patients with active BrM, while the larger phase 3b/4 DESTINY-Breast12 trial reported an intracranial response rate of 62.3% overall, rising to 82.6% in patients with untreated BrM [20, 21]. In HR+ disease, the CDK4/6 inhibitor abemaciclib has demonstrated blood-brain barrier penetration and clinical activity in patients with BrM, expanding treatment options beyond traditional endocrine therapy and supporting a role for CNS-active systemic management in this subtype [22]. In triple-negative breast cancer, sacituzumab govitecan has also demonstrated clinically meaningful survival benefit and emerging CNS activity [23]. More recently, datopotamab deruxtecan has shown promising efficacy in heavily pretreated HR + and triple-negative MBC and may further expand treatment options for patients with BrM, although prospective intracranial efficacy data remain limited [24]. Collectively, these therapies represent a paradigm shift in the management of breast cancer with BrM, with increasing capacity for durable intracranial disease control using systemic therapy. As CNS-penetrant therapies continue to improve extracranial and intracranial disease control, the relative importance of optimizing local CNS-directed strategies, minimizing long-term neurotoxicity, and understanding distinct metastatic phenotypes such as BO-MBC will likely become increasingly clinically relevant.

Within BO-MBC, SRS/SRT was associated with improved OS compared with WBRT; however, this finding should be interpreted with substantial caution and should not be considered evidence of definitive modality superiority. Multiple randomized trials comparing SRS/SRT and WBRT for BrM have demonstrated no consistent OS advantage with SRS/SRT alone, with the principal benefit of stereotactic approaches relating instead to preservation of neurocognitive function and quality of life [25, 26]. In our analysis, the OS advantage associated with SRS/SRT likely reflects treatment selection bias and residual confounding rather than a direct treatment effect. Critically, the NCDB does not capture several major determinants of radiotherapy selection and prognosis, including intracranial disease burden, number and volume of BrM, lesion location, neurologic symptoms, performance status, extracranial disease control, salvage intracranial therapies, or LMD progression. Patients selected for SRS/SRT in routine clinical practice are therefore more likely to have favorable prognostic characteristics, lower intracranial disease burden, and better functional status compared with patients receiving WBRT. Accordingly, minimizing long-term neurotoxicity becomes increasingly important in BO-MBC patients who may experience prolonged survival with CNS-dominant disease. In this context, neurocognitive-sparing stereotactic strategies integrated with effective systemic therapy may represent an important component of multidisciplinary management for appropriately selected patients.

This study has limitations inherent to registry analyses. NCDB lacks key CNS variables (number and size of metastases, symptoms, edema, steroid dependence, performance status, salvage treatments, and LMD), precluding adjustment for major determinants of radiotherapy selection and limiting causal inference, particularly for WBRT vs. SRS/SRT comparisons. Although the NCDB includes an aggregate variable for surgery of other regional or distant sites, it does not identify the operative site and therefore cannot distinguish neurosurgical resection of brain metastases from surgery of extracranial metastatic disease. Consequently, neurosurgical resection could not be evaluated or adjusted for, potentially contributing to residual confounding in the OS analyses because surgical candidates may have more favorable clinical characteristics and limited disease burden. Treatment coding also does not reliably capture detailed radiotherapy dose, fractionation intent, or technical nuances of intracranial treatment, such as distinction between single-fraction SRS, hypofractionated SRT, or stereotactic boost following WBRT, nor does it reliably specify contemporary systemic regimens with meaningful CNS activity. Additionally, systemic therapy variables are limited to documented first-course treatment, may incompletely capture therapy administered outside the reporting facility, and do not capture subsequent-line therapy; therefore, the proportion of patients classified as not receiving systemic therapy should be interpreted cautiously. Misclassification of metastatic pattern is also possible, particularly if ECM was occult or underreported. In addition, the institutional cohort is limited by small sample size and single-institution practice patterns. The institutional cohort also lacked a contemporaneous BrM + ECM comparator with equivalent longitudinal curation. Consequently, CNS-specific outcomes could not be directly compared between metastatic phenotypes at the institutional level, and comparisons between BO-MBC and BrM + ECM relied on variables available in the NCDB. Furthermore, given the small institutional cohort and the overlap between intracranial and extracranial progression events, we could not reliably evaluate clinicopathologic or treatment factors associated with patterns of progression. These relationships should be investigated in larger cohorts. However, the institutional cohort provides clinically important granularity regarding progression dynamics and treatment-related toxicity that is unavailable in national datasets. Key strengths include a large contemporary national cohort, prespecified subgroup and interaction analyses, rigorous covariate balancing using overlap weighting, and integration of population-level findings with institutional CNS-specific outcomes.

In conclusion, BO-MBC may represent a distinct subgroup with unique clinicopathologic features, treatment patterns, and improved OS compared with BrM + ECM. Among patients with BO-MBC receiving brain radiotherapy, SRS/SRT was associated with improved OS relative to WBRT, although this finding likely reflects treatment selection and unmeasured intracranial disease burden. Institutional data further demonstrate durable extracranial control, while intracranial progression remained the predominant failure pattern, underscoring the need for integrated multidisciplinary management strategies that prioritize optimized stereotactic approaches, CNS surveillance, and contemporary systemic therapy.

## Supplementary Information

Below is the link to the electronic supplementary material.

Supplementary Material 1

## Data Availability

NCDB data are available through the American College of Surgeons and the Commission on Cancer. Institutional data is available from the corresponding author upon reasonable request.
