# Supplementary material for: Brain-only metastatic breast cancer as a distinct clinical entity: evidence from national and institutional cohorts with implications for radiation therapy
Source: Breast Cancer Res Treat. 2026 Oct 7;219(3):29. doi: 10.1007/s10549-026-08092-3 (PMC13645911; doi:10.1007/s10549-026-08092-3)
Supplement: Supplementary file 1 [file 10549_2026_8092_MOESM1_ESM.docx]

| Supplemental Table 1 - Sociodemographic, Clinical and Treatment Characteristics Comparing BO-MBC to BrM+ECM Group Type (n=8,909) | | | | | | |
| --- | --- | --- | --- | --- | --- | --- |
|  | **Unweighted** | | | **Weighted^b^** | | |
|  | **BO-MBC (n=1540)** | **BrM+ECM (n=7369)** | **Standardize Difference^a^** | **BO-MBC (n=1540)** | **BrM+ECM (n=7369)** | **Standardize Difference^a^** |
| **Age** |  |  | 0.0932 |  |  | 0 |
| Median (IQR) | 62 (52, 71) | 61 (53, 69) |  | 61 (52, 71) | 62 (53, 70) |  |
|  |  |  |  |  |  |  |
| **Race**, n (%) |  |  |  |  |  |  |
| White | 1142 (75.3%) | 5625 (76.9%) | 0.0384 | 75.6% | 75.6% | 0 |
| African American/Black | 298 (19.6%) | 1340 (18.3%) | 0.0337 | 19.4% | 19.4% | 0 |
| Asian | 50 (3.3%) | 224 (3.1%) | 0.0133 | 3.3% | 3.3% | 0 |
| Other | 27 (1.8%) | 124 (1.7%) | 0.0064 | 1.7% | 1.7% | 0 |
|  |  |  |  |  |  |  |
| **Ethnicity,** n (%) |  |  |  |  |  |  |
| Non-Hispanic | 1361 (91.3%) | 6650 (92.5%) | 0.0431 | 91.8% | 91.8% | 0 |
| Hispanic | 129 (8.7%) | 538 (7.5%) | 0.0431 | 8.2% | 8.2% | 0 |
|  |  |  |  |  |  |  |
| **Charlson-Deyo Score**, n (%) |  |  |  |  |  |  |
| 0 | 1247 (81.0%) | 5886 (79.9%) | 0.0277 | 80.5% | 80.5% | 0 |
| 1 | 174 (11.3%) | 1010 (13.7%) | 0.0728 | 11.7% | 11.7% | 0 |
| 2 | 70 (4.5%) | 291 (3.9%) | 0.0296 | 4.6% | 4.6% | 0 |
| 3+ | 49 (3.2%) | 182 (2.5%) | 0.0430 | 3.1% | 3.1% | 0 |
|  |  |  |  |  |  |  |
| **Insurance**, n (%) |  |  |  |  |  |  |
| Not Insured | 101 (6.7%) | 541 (7.5%) | 0.0294 | 6.9% | 7.4% | 0.0187 |
| Private Insurance | 552 (36.8%) | 2742 (37.9%) | 0.0240 | 36.9% | 36.4% | 0.0102 |
| Medicaid | 230 (15.3%) | 1188 (16.4%) | 0.0304 | 15.5% | 15.7% | 0.0064 |
| Medicare | 612 (40.7%) | 2696 (37.3%) | 0.0712 | 40.2% | 39.7% | 0.0116 |
| Other Government | 7 (0.5%) | 66 (0.9%) | 0.0540 | 0.5% | 0.8% | 0.0431 |
|  |  |  |  |  |  |  |
| **Median Income Quartiles**, n (%) |  |  |  |  |  |  |
| <$46,227 | 283 (18.4%) | 1230 (16.7%) | 0.0443 | 18.1% | 16.9% | 0.0317 |
| $46,227-$57,856 | 299 (19.4%) | 1507 (20.5%) | 0.0259 | 19.4% | 20.4% | 0.0250 |
| $57,587-$74,062 | 329 (21.4%) | 1468 (19.9%) | 0.0356 | 21.3% | 20.1% | 0.0285 |
| $74,063 or higher | 404 (26.2%) | 2178 (29.6%) | 0.0741 | 26.3% | 28.8% | 0.0556 |
| Unknown | 225 (14.6%) | 986 (13.4%) | 0.0355 | 14.9% | 13.8% | 0.0321 |
|  |  |  |  |  |  |  |
| **Education**, n (%) |  |  |  |  |  |  |
| 15.3% or higher | 333 (21.6%) | 1586 (21.5%) | 0.0025 | 21.3% | 22.0% | 0.0170 |
| 9.1%-15.2% | 408 (26.5%) | 1885 (25.6%) | 0.0208 | 26.4% | 25.7% | 0.0162 |
| 5.0%-9.0% | 346 (22.5%) | 1783 (24.2%) | 0.0409 | 22.5% | 23.8% | 0.0329 |
| <5.0% | 236 (15.3%) | 1144 (15.5%) | 0.0055 | 15.5% | 14.9% | 0.0156 |
| Unknown | 217 (14.1%) | 971 (13.2%) | 0.0266 | 14.4% | 13.6% | 0.0235 |
|  |  |  |  |  |  |  |
| **Community**, n (%) |  |  |  |  |  |  |
| Rural | 17 (1.1%) | 117 (1.6%) | 0.0422 | 1.1% | 1.7% | 0.0488 |
| Urban | 199 (13.3%) | 884 (12.3%) | 0.0298 | 13.3% | 12.2% | 0.0320 |
| Metro | 1277 (85.5%) | 6168 (86.0%) | 0.0145 | 85.6% | 86.1% | 0.0141 |
|  |  |  |  |  |  |  |
| **Nodal**, n (%) |  |  |  |  |  |  |
| 0 | 439 (28.5%) | 1480 (20.1%) | 0.1973 | 28.3% | 20.3% | 0.1889 |
| 1 | 495 (32.1%) | 2803 (38.0%) | 0.1238 | 32.3% | 37.7% | 0.1125 |
| 2 | 123 (8.0%) | 740 (10.0%) | 0.0718 | 8.0% | 9.7% | 0.0567 |
| 3 | 208 (13.5%) | 952 (12.9%) | 0.0174 | 13.7% | 13.3% | 0.0102 |
| Unknown | 275 (17.9%) | 1394 (18.9%) | 0.0274 | 17.6% | 19.1% | 0.0374 |
|  |  |  |  |  |  |  |
| **Grade**, n (%) |  |  |  |  |  |  |
| 1 | 73 (4.7%) | 301 (4.1%) | 0.0319 | 4.8% | 3.7% | 0.0552 |
| 2 | 274 (17.8%) | 1652 (22.4%) | 0.1156 | 18.2% | 20.1% | 0.0475 |
| 3 | 508 (33.0%) | 2250 (30.5%) | 0.0527 | 33.1% | 32.9% | 0.0041 |
| Unknown | 685 (44.5%) | 3166 (43.0%) | 0.0306 | 43.9% | 43.4% | 0.0115 |
|  |  |  |  |  |  |  |
| **Subtype**, n (%) |  |  |  |  |  |  |
| ER+/PR+/HER2- | 342 (22.2%) | 2305 (31.3%) | 0.2060 | 23.7% | 23.7% | 0 |
| ER+/PR (+ or -)/HER2+ | 176 (11.4%) | 1014 (13.8%) | 0.0703 | 11.8% | 11.8% | 0 |
| ER-/PR-/HER2+ | 173 (11.2%) | 765 (10.4%) | 0.0275 | 11.0% | 11.0% | 0 |
| ER-/PR-/HER2- | 400 (26.0%) | 1305 (17.7%) | 0.2010 | 24.6% | 24.6% | 0 |
| Unknown | 449 (29.2%) | 1980 (26.9%) | 0.0509 | 28.9% | 28.9% | 0 |
|  |  |  |  |  |  |  |
| **Treatment facility type**, n (%) |  |  |  |  |  |  |
| Community Cancer Program | 113 (7.8%) | 481 (7.0%) | 0.0320 | 7.7% | 6.9% | 0.0314 |
| Comprehensive Community Cancer Program | 542 (37.5%) | 2622 (38.0%) | 0.0114 | 37.3% | 38.1% | 0.0169 |
| Academic/Research Program | 508 (35.1%) | 2365 (34.3%) | 0.0173 | 35.2% | 34.4% | 0.0164 |
| Integrated Network Cancer Program | 284 (19.6%) | 1430 (20.7%) | 0.0275 | 19.8% | 20.6% | 0.0195 |
|  |  |  |  |  |  |  |
| **Systemic Therapy**, n (%) |  |  |  |  |  |  |
| Yes | 1033 (67.2%) | 5483 (74.5%) | 0.1614 | 68.7% | 68.7% | 0 |
| No | 504 (32.8%) | 1875 (25.5%) | 0.1614 | 31.3% | 31.3% | 0 |
| **Brain Radiation**, n (%) |  |  |  |  |  |  |
| Yes | 844 (54.8%) | 3020 (41.0%) | 0.2794 | 53.0% | 53.0% | 0 |
| No | 696 (45.2%) | 4349 (59.0%) | 0.2794 | 47.0% | 47.0% | 0 |

^a^ Standardized differences = absolute difference in means or proportions divided by pooled standard deviation. Values >0.10 suggest imbalance; whereas smaller values indicated better balance. Values of 0 suggest exact balance for that variable.

^b^ Overlap Propensity Score Weighting was used to account for confounding due to differences in baseline characteristics that may affect receipt of group type. Weights were adjusted using age, race, ethnicity, Charlson-Deyo score, molecular subtype, systemic therapy and brain radiotherapy.

| Supplemental Table 2 - Sociodemographic, Clinical and Treatment Characteristics Comparing SRS/SRT to WBRT Brain Radiotherapy (n=3,864) | | | | | | |
| --- | --- | --- | --- | --- | --- | --- |
|  | **Unweighted** | | | **Weighted^b^** | | |
|  | **WBRT**  **(n=2,863)** | **SRS/SRT**  **(n=1,001)** | **Standardize Difference^a^** | **WBRT**  **(n=2,863)** | **SRS/SRT**  **(n=1,001)** | **Standardize Difference^a^** |
| **Age** |  |  | 0.0606 |  |  | 0 |
| Median (IQR) | 60 (52, 68) | 59 (51, 67) |  | 61 (53, 69) | 61 (53, 68) |  |
|  |  |  |  |  |  |  |
| **Race**, n (%) |  |  |  |  |  |  |
| White | 2166 (76.3%) | 780 (78.5%) | 0.0533 | 79.0% | 79.0% | 0 |
| African American/Black | 551 (19.4%) | 163 (16.4%) | 0.0783 | 16.7% | 16.7% | 0 |
| Asian | 72 (2.5%) | 39 (3.9%) | 0.0787 | 3.3% | 3.3% | 0 |
| Other | 49 (1.7%) | 11 (1.1%) | 0.0524 | 1.0% | 1.0% | 0 |
|  |  |  |  |  |  |  |
| **Ethnicity,** n (%) |  |  |  |  |  |  |
| Non-Hispanic | 2596 (93.1%) | 912 (92.4%) | 0.0262 | 93.6% | 93.6% | 0 |
| Hispanic | 193 (6.9%) | 75 (7.6%) | 0.0262 | 6.4% | 6.4% | 0^e^ |
|  |  |  |  |  |  |  |
| **Charlson-Deyo Score**, n (%) |  |  |  |  |  |  |
| 0 | 2293 (80.1%) | 825 (82.4%) | 0.0596 | 81.0% | 81.0% | 0 |
| 1 | 391 (13.7%) | 114 (11.4%) | 0.0686 | 12.3% | 12.3% | 0 |
| 2 | 107 (3.7%) | 45 (4.5%) | 0.0382 | 4.6% | 4.6% | 0 |
| 3+ | 72 (2.5%) | 17 (1.7%) | 0.0569 | 2.1% | 2.1% | 0 |
|  |  |  |  |  |  |  |
| **Insurance**, n (%) |  |  |  |  |  |  |
| Not Insured | 207 (7.4%) | 54 (5.4%) | 0.0783 | 5.9% | 5.9% | 0 |
| Private Insurance | 1132 (40.2%) | 421 (42.4%) | 0.0444 | 40.3% | 40.3% | 0 |
| Medicaid | 453 (16.1%) | 181 (18.2%) | 0.0567 | 16.5% | 16.5% | 0 |
| Medicare | 1003 (35.6%) | 331 (33.3%) | 0.0483 | 36.6% | 36.6% | 0 |
| Other Government | 20 (0.7%) | 6 (0.6%) | 0.0131 | 0.6% | 0.6% | 0 |
|  |  |  |  |  |  |  |
| **Median Income Quartiles**, n (%) |  |  |  |  |  |  |
| <$46,227 | 521 (18.2%) | 145 (14.5%) | 0.1005 | 16.6% | 13.4% | 0.0906 |
| $46,227-$57,856 | 589 (20.6%) | 180 (18.0%) | 0.0657 | 19.5% | 18.8% | 0.0191 |
| $57,587-$74,062 | 593 (20.7%) | 221 (22.1%) | 0.0333 | 21.5% | 22.4% | 0.0227 |
| $74,063 or higher | 756 (26.4%) | 307 (30.7%) | 0.0945 | 27.9% | 31.2% | 0.0724 |
| Unknown | 404 (14.1%) | 148 (14.8%) | 0.0192 | 14.4% | 14.1% | 0.0074 |
|  |  |  |  |  |  |  |
| **Education**, n (%) |  |  |  |  |  |  |
| 15.3% or higher | 594 (20.7%) | 205 (20.5%) | 0.0066 | 19.8% | 19.0% | 0.0208 |
| 9.1%-15.2% | 771 (26.9%) | 247 (24.7%) | 0.0515 | 25.7% | 25.1% | 0.0152 |
| 5.0%-9.0% | 692 (24.2%) | 219 (21.9%) | 0.0545 | 24.7% | 23.4% | 0.0314 |
| <5.0% | 411 (14.4%) | 187 (18.7%) | 0.1167 | 15.5% | 18.9% | 0.0887 |
| Unknown | 395 (13.8%) | 143 (14.3%) | 0.0141 | 14.2% | 13.7% | 0.0150 |
|  |  |  |  |  |  |  |
| **Community**, n (%) |  |  |  |  |  |  |
| Rural | 43 (1.6%) | 19 (2.0%) | 0.0318 | 1.8% | 1.8% | 0 |
| Urban | 395 (14.2%) | 105 (10.9%) | 0.1016 | 12.2% | 12.2% | 0 |
| Metro | 2335 (84.2%) | 841 (87.2%) | 0.0842 | 86.0% | 86.0% | 0 |
|  |  |  |  |  |  |  |
| **Nodal**, n (%) |  |  |  |  |  |  |
| 0 | 599 (20.9%) | 212 (21.2%) | 0.0063 | 20.7% | 21.4% | 0.0155 |
| 1 | 1057 (36.9%) | 391 (39.1%) | 0.0441 | 36.9% | 38.5% | 0.0325 |
| 2 | 301 (10.5%) | 106 (10.6%) | 0.0025 | 10.8% | 10.5% | 0.0098 |
| 3 | 421 (14.7%) | 157 (15.7%) | 0.0273 | 15.0% | 15.8% | 0.0238 |
| Unknown | 485 (16.9%) | 135 (13.5%) | 0.0963 | 16.6% | 13.8% | 0.0770 |
|  |  |  |  |  |  |  |
| **Grade**, n (%) |  |  |  |  |  |  |
| 1 | 120 (4.2%) | 31 (3.1%) | 0.0584 | 4.2% | 3.3% | 0.0498 |
| 2 | 577 (20.2%) | 204 (20.4%) | 0.0056 | 20.8% | 20.6% | 0.0047 |
| 3 | 1023 (35.7%) | 358 (35.8%) | 0.0007 | 35.2% | 35.0% | 0.0032 |
| Unknown | 1143 (39.9%) | 408 (40.8%) | 0.0170 | 39.8% | 41.4% | 0.0264 |
|  |  |  |  |  |  |  |
| **Subtype**, n (%) |  |  |  |  |  |  |
| ER+/PR+/HER2- | 724 (25.3%) | 288 (28.8%) | 0.0785 | 28.7% | 28.7% | 0 |
| ER+/PR (+ or -)/HER2+ | 424 (14.8%) | 147 (14.7%) | 0.0035 | 13.6% | 13.6% | 0 |
| ER-/PR-/HER2+ | 365 (12.7%) | 127 (12.7%) | 0.0018 | 12.8% | 12.8% | 0 |
| ER-/PR-/HER2- | 693 (24.2%) | 200 (20.0%) | 0.1020 | 20.7% | 20.7% | 0 |
| Unknown | 657 (22.9%) | 239 (23.9%) | 0.0219 | 24.3% | 24.3% | 0 |
|  |  |  |  |  |  |  |
| **Treatment facility type**, n (%) |  |  |  |  |  |  |
| Community Cancer Program | 196 (7.3%) | 43 (4.6%) | 0.1114 | 5.2% | 5.2% | 0 |
| Comprehensive Community Cancer Program | 1054 (39.2%) | 323 (34.9%) | 0.0881 | 35.7% | 35.7% | 0 |
| Academic/Research Program | 885 (32.9%) | 374 (40.4%) | 0.1571 | 38.7% | 38.7% | 0 |
| Integrated Network Cancer Program | 556 (20.7%) | 185 (20.0%) | 0.0164 | 20.5% | 20.5% | 0 |
|  |  |  |  |  |  |  |
| **Chemotherapy**, n (%) |  |  |  |  |  |  |
| Yes | 1787 (63.5%) | 698 (70.7%) | 0.1545 | 67.4% | 67.4% | 0 |
| No | 1028 (36.5%) | 289 (29.3%) | 0.1545 | 32.6% | 32.6% | 0 |
|  |  |  |  |  |  |  |
| **Hormone therapy**, n (%) |  |  |  |  |  |  |
| Yes | 1082 (38.4%) | 425 (43.2%) | 0.0977 | 42.6% | 42.6% | 0 |
| No | 1733 (61.6%) | 558 (56.8%) | 0.0977 | 57.4% | 57.4% | 0 |
|  |  |  |  |  |  |  |
| **Immunotherapy**, n (%) |  |  |  |  |  |  |
| Yes | 595 (20.9%) | 267 (26.8%) | 0.1377 | 25.1% | 25.1% | 0 |
| No | 2252 (79.1%) | 731 (73.2%) | 0.1377 | 74.9% | 74.9% | 0 |

^a^ Standardized differences = absolute difference in means or proportions divided by pooled standard deviation. Values >0.10 suggest imbalance; whereas smaller values indicated better balance. Values of 0 suggest exact balance for that variable.

^b^ Overlap Propensity Score Weighting was used to account for confounding due to differences in baseline characteristics that may affect receipt of brain radiotherapy type. Weights were adjusted using age, race, ethnicity, Charlson-Deyo score, insurance, community, molecular subtype, facility type, chemeotherapy, hormone therapy and immunotherapy.

*Abbreviations*: WBRT=Whole brain radiation therapy; SRS=Stereotactic radiosurgery

Supplemental Table 3. Patient Characteristics Comparing Whole-Brain Radiation Therapy and Stereotactic Radiosurgery in the NCDB Cohort (n = 3,864)

| **Variable** | **WBRT (n = 2,863)** | **SRS/SRT (n = 1,001)** | **P-value** |
| --- | --- | --- | --- |
| **Age**, median (IQR) | 60 (52-68) | 59 (51-67) | 0.075 |
|  |  |  |  |
| **Race, n (%)** |  |  | 0.013 |
| White | 2166 (76.3%) | 780 (78.5%) |  |
| Black | 551 (19.4%) | 163 (16.4%) |  |
| Asian | 72 (2.5%) | 39 (3.9%) |  |
| Other | 49 (1.7%) | 11 (1.1%) |  |
|  |  |  |  |
| **Ethnicity, n (%)** |  |  | 0.475 |
| Non-Hispanic | 2596 (93.1%) | 912 (92.4%) |  |
| Hispanic | 193 (6.9%) | 75 (7.6%) |  |
|  |  |  |  |
| **Charlson-Deyo score, n (%)** |  |  | 0.084 |
| 0 | 2293 (80.7%) | 825 (82.4%) |  |
| 1 | 391 (13.7%) | 114 (11.4%) |  |
| 2 | 107 (3.7%) | 45 (4.5%) |  |
| 3+ | 72 (2.5%) | 17 (1.7%) |  |
|  |  |  |  |
| **Insurance, n (%)** |  |  | 0.091 |
| Uninsured | 207 (7.4%) | 54 (5.4%) |  |
| Private | 1132 (40.2%) | 421 (42.4%) |  |
| Medicaid | 453 (16.1%) | 181 (18.2%) |  |
| Medicare | 1003 (35.6%) | 331 (33.3%) |  |
| Other government | 20 (0.7%) | 6 (0.6%) |  |
|  |  |  |  |
| **Facility type, n (%)** |  |  | <0.001 |
| Community Cancer Program | 196 (7.3%) | 43 (4.6%) |  |
| Comprehensive Community Program | 1054 (39.2%) | 323 (34.9%) |  |
| Academic/Research Program | 885 (32.9%) | 374 (40.4%) |  |
| Integrated Network Program | 556 (20.7%) | 185 (20.0%) |  |
|  |  |  |  |
| **Subtype, n (%)** |  |  | 0.051 |
| HR+/HER2- | 724 (25.3%) | 288 (28.8%) |  |
| HR+/HER2+ | 424 (14.8%) | 147 (14.7%) |  |
| HR-/HER2+ | 365 (12.7%) | 127 (12.7%) |  |
| Triple negative | 693 (24.2%) | 200 (20.0%) |  |
| Unknown | 657 (22.9%) | 239 (23.9%) |  |
|  |  |  |  |
| **Systemic therapy, n (%)** |  |  | <0.001 |
| Yes | 2321 (81.1%) | 867 (86.8%) |  |
| No | 540 (18.9%) | 132 (13.2%) |  |
| Includes only patients receiving brain radiotherapy in the NCDB cohort. | | | |
